# Application and challenges of DeepSeek in primary care in China

**DOI:** 10.3389/fpubh.2025.1721308

**Published:** 2025-11-28

**Authors:** Jinyi Zhang, Dong Li, Xingyou Wang, Zengxiang Wu, Qingguo Lyu

**Affiliations:** 1 West China School of Medicine, Sichuan University, Sichuan University Affiliated Chengdu Second People's Hospital, Chengdu Second People's Hospital, Chengdu, China; 2 Department of Computing, The Hong Kong Polytechnic University, Kowloon, China; 3 Department of Oncology, Ziyang Central Hospital, Ziyang, China; 4 School of Health Policy and Management, Chinese Academy of Medical Sciences and Peking Union Medical College, Beijing, China; 5 General Practice Ward/International Medical Center Ward, General Practice Medical Center, National Clinical Research Center for Geriatrics, West China Hospital, Sichuan University, Chengdu, China; 6 Department of Endocrinology and Metabolism, West China Hospital, Sichuan University, Chengdu, China

**Keywords:** DeepSeek, primary care, artificial intelligence, clinical decision support, medical education

## Abstract

China’s primary care system faced persistent challenges, including uneven resource distribution, a shortage of general practitioners, and a growing burden of chronic diseases. Artificial intelligence (AI) offered new tools to address these issues. This narrative review summarized the applications, benefits, challenges, and practical recommendations for DeepSeek. Literature searches were conducted across both Chinese and English databases, including China National Knowledge Infrastructure, Wanfang Data, and PubMed. In addition, official websites of provincial Health Commissions were searched for AI policies and reports related to DeepSeek deployment. Evidence showed that DeepSeek had been applied to assist clinical decision-making, support chronic disease management, and enhance medical education and research. Reported outcomes included improved diagnostic efficiency, guideline adherence, and patient engagement. However, challenges remained, such as limited model interpretability, potential reductions in humanistic care, unequal accessibility, technical constraints, and data privacy concerns.

## 1 Background

China faced uneven healthcare resource distribution, a shortage of general practitioners (GPs), and a growing burden of chronic disease (1, 2). Primary care institutions in China constituted the most widely distributed healthcare facilities (3). They delivered essential public health services, routine medical care, and chronic disease management to the majority of the population (3). However, they often faced limited specialty capabilities, particularly for vulnerable populations such as the older adults and children (4). Workforce shortages, insufficient equipment, and gaps in clinical experience hindered the delivery of high-quality care (5). Chronic diseases—including hypertension, type 2 diabetes, and malignancies—continued to rise (6). They contributed significantly to national morbidity and mortality and imposed substantial burdens on primary care providers (6).

Artificial intelligence (AI) offered novel approaches to support primary care and rapidly permeated clinical practice, pharmaceuticals, and health management, driving significant transformation across the healthcare system (7). AI demonstrated potential to address these challenges. It enhanced diagnostic accuracy, supported clinical decision-making, and streamlined workflow in primary care (7, 8). AI-powered systems integrated multi-source data, including electronic health records, laboratory tests, and wearable device measurements. They identified high-risk individuals, guided preventive interventions, and monitored treatment outcomes in real time (9). Early implementations in community hospitals and pilot studies showed that AI assistance improved guideline adherence, optimized chronic disease management, and reduced clinician workload (10).

In January 2025, Hangzhou Deeply Seeking Artificial Intelligence Basic Technology Research Co., Ltd. released DeepSeek-R1, a reasoning-optimized large language model based on DeepSeek-V3 and designed for complex inferential tasks (11). Many primary care settings in China adopted DeepSeek models to enhance clinical practice (12). This narrative review examined the benefits and challenges of DeepSeek across clinical practice, research, and education, highlighting lessons for the effective adoption of generative AI in China’s primary healthcare system.

## 2 Methods

**Figure 1:**
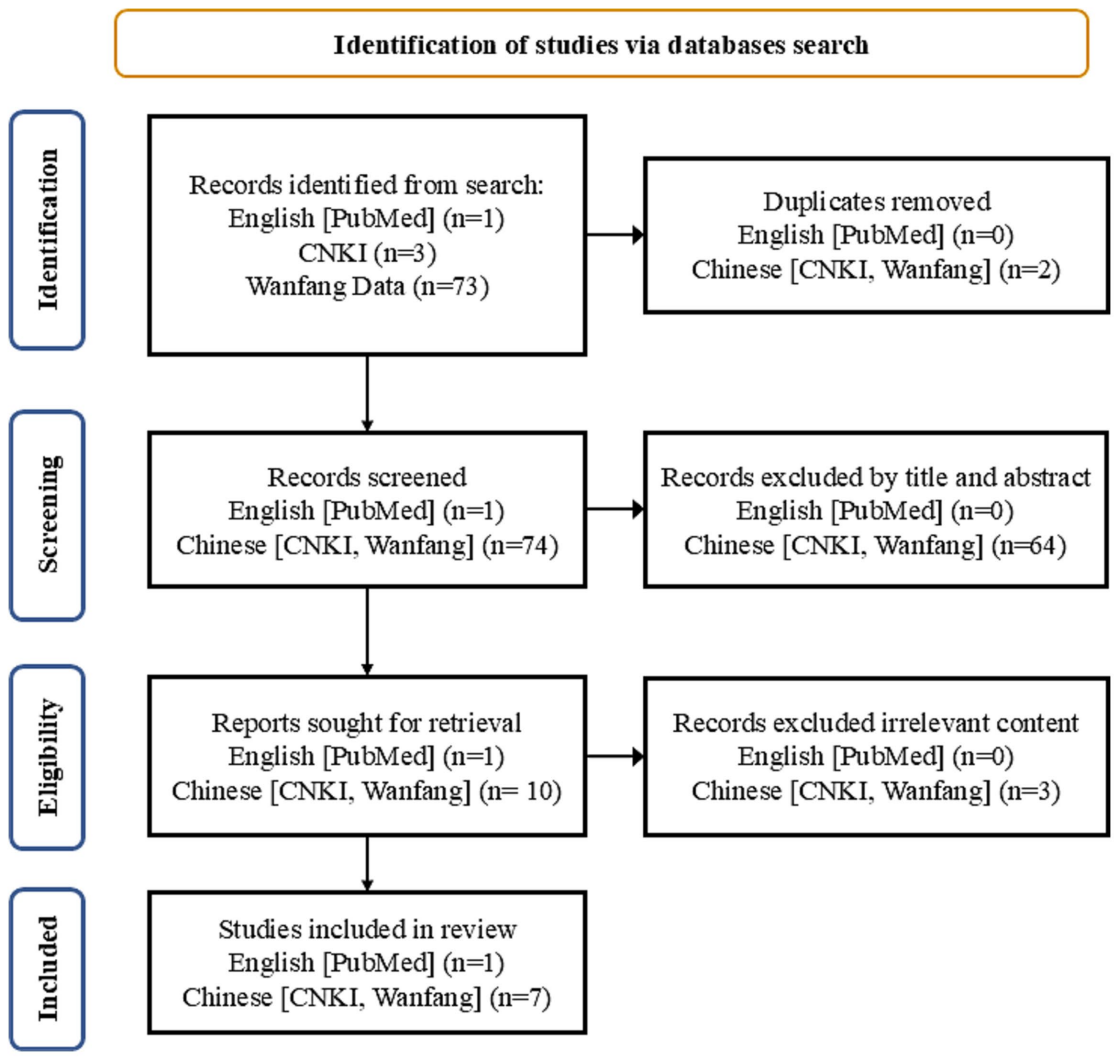
Flow diagram depicting the strategy for literature search and selection.

Searches were conducted in both Chinese and English databases, including China National Knowledge Infrastructure (CNKI), Wanfang Data, and PubMed. Primary search terms included ‘DeepSeek,’ ‘artificial intelligence,’ ‘primary care,’ ‘general practitioners,’ and MeSH term ‘Clinical Decision Support Systems.’ The search covered articles published between January 1, 2023, and April 14, 2025, reflecting the period when AI-based clinical decision tools were increasingly adopted in China. Inclusion criteria were: (1) publications in English or Chinese; (2) articles or reviews; (3) studies focused on the use of DeepSeek in primary care. Exclusion criteria were: (1) studies focused on medical education; (2) conference papers, theses, commentaries, editorials, and letters. Details of the literature search and screening process are presented in Figure 1. Furthermore, official websites of provincial Health Commissions were searched for AI policies and reports related to DeepSeek deployment.

## 3 Results

### 3.1 Chinese AI policy

China has been actively developing regulatory frameworks for AI, particularly generative AI. Key policies included the *Algorithm Recommendation Management Provisions* (2021) (13), the *Deep Synthesis Management Provisions for Internet Information Services* (2022) (14), the *Interim Measures for the Management of Generative AI Services* (2023) (15), and the *Measures for the Identification of AI-Generated Content* (2025) (16). The *Measures for Ethical Review of Scientific and Technological Activities* (Trial) (2023) mandate ethical assessment for algorithms with social influence (17). these regulations aim to promote the ethical and socially responsible use of AI while safeguarding data security, ensuring transparent content labeling, and defining the obligations of service providers.

### 3.2 DeepSeek technical overview

**Figure 2:**
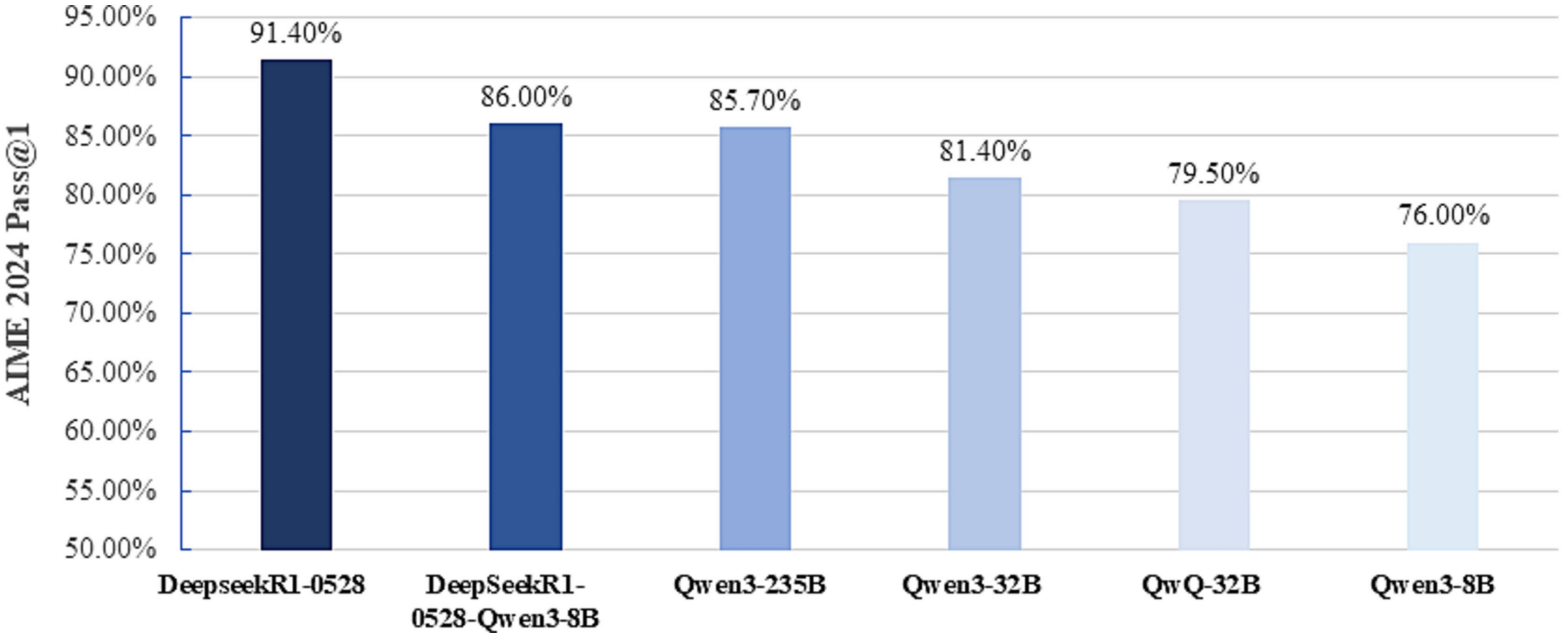
Performance of Chinese large language models on the 2024 American Invitational Mathematics Examination. AIME, American Invitational Mathematics Examination;pass@1, proportion of correct answers at the first attempt (%).

DeepSeek is an open-source platform licensed under the Massachusetts Institute of Technology (MIT) framework, providing a cost-effective alternative to proprietary AI systems (18). Its exceptional inferential capabilities derive from a Mixture-of-Experts architecture, reinforcement learning from human feedback (RLHF), an FP8 mixed-precision framework, and a multi-stage training regimen (18). In benchmark evaluations, DeepSeek-R1 outperformed both Generative Pre-trained Transformer (GPT-4o) and OpenAI’s o1 reasoning model (see Table 1) (19). Its capabilities extended into multimodal AI with the Janus-Pro-7B variant (11). DeepSeek demonstrated superior performance compared with other Chinese AI models (see Figure 2) (20).

**Table 1:** Performance comparison of leading large language models across standardized benchmarks (pass@1, %).

Benchmark	DeepSeek-R1-0528	OpenAI-o3	Gemini-2.5-Pro-0506	Qwen3-235B	DeepSeek-R1
AIME 2024 Mathematics Competition	91.4	91.6	90.8	85.7	79.8
AIME 2025 Mathematics Competition	87.5	88.9	83.0	81.5	70.0
GPQA Diamond (Scientific Reasoning)	81.0	83.3	83.0	71.1	71.5
LiveCodeBench (Code Generation)	73.3	77.3	71.8	66.5	63.5
Aider (Code Editing)	71.6	79.6	76.9	65.0	57.0
Humanity’s Last Exam (Reasoning and Knowledge)	17.7	20.6	18.4	11.8	8.5

AIME, American Invitational Mathematics Examination; GPQA, Graduate-level Google-Proof Q&A; pass@1, proportion of correct answers at the first attempt (%).

The model’s deep reasoning ability enables support for general practitioners in clinical practice, research, and educational training, particularly when augmented with high-quality, up-to-date knowledge during fine-tuning (21). DeepSeek offers flexibility via an application programming interface (API), allowing users to access its functions at lower cost than other large models and to run it locally (11). Unlike many general-purpose models, which often lack detailed version histories and transparent update logs, DeepSeek employs a dual identifier system of model version and training timestamp (22). During fine-tuning, all parameter adjustments are fully recorded, including supervised fine-tuning (SFT) datasets and RLHF ethical alignment strategies, ensuring traceability and accountability in model updates (18).

### 3.3 Advantages of DeepSeek

DeepSeek-R1 leveraged existing AI tools, including Mixture-of-Experts (MoE), reinforcement learning, and data distillation, to achieve breakthrough reasoning efficiency (18). It uniquely revealed the “chain of thought” during inference, enhancing transparency for users (see Table 2) (18). By deploying smaller, reliable, and open models on flexible infrastructures, the platform ensured effective and safe AI integration. DeepSeek strictly followed TRIPOD-LLM reporting guidelines, enabling full traceability from data de-identification to decision annotation, and establishing an ethically grounded technical framework (11, 12, 18).

**Table 2:** Key advantages of DeepSeek.

Advantage	Description
Capability to handle complex medical data	DeepSeek effectively processed heterogeneous healthcare data, including images, clinical notes, and structured numerical values. It accurately interpreted medical terminology and complex disease descriptions.
Strong generalization ability	Unlike task-specific models, DeepSeek adapted to diverse medical tasks, such as disease detection, prognosis evaluation, and patient risk prediction, via its Mixture-of-Experts architecture.
Real-time data processing	The model supported real-time analysis and prediction using continuously updated clinical data. This capability was particularly valuable for decision support in time-sensitive clinical environments.
Personalized medicine	By learning from large-scale patient datasets, DeepSeek generated individualized medical insights and treatment recommendations. This enabled more patient-centered and precise healthcare delivery.

### 3.4 Applications of DeepSeek in primary care

#### 3.4.1 Empowering general practitioners

In primary care settings, diagnostic accuracy and timely decision-making were critical. DeepSeek assisted GPs by automating repetitive tasks, including standardized data entry, intelligent retrieval and integration of dispersed medical information, and generation of structured clinical documentation (23). The platform integrated multidimensional patient data—such as medical history, physical findings, and test results—and applied evidence-based reasoning to generate clinical recommendations (12). Logical inference modules reduced purely data-driven biases and supported personalized treatment planning, improving healthcare quality and outcomes (18). In a recent survey of GPs in Zhejiang Province, 64.3% reported actively using DeepSeek, and more than half (54.4%) perceived it as a collaborative decision-making tool rather than a replacement for clinicians (24).

A representative clinical case further illustrated the promise of DeepSeek-assisted diagnosis (25). A 57-year-old woman with recurrent epigastric pain consulted multiple surgeons and underwent extensive gastrointestinal and cardiologic workups, which yielded nonspecific findings. Dissatisfied with persistent symptoms, she subsequently engaged in more than 100 rounds of interaction with the DeepSeek system. The model suggested a possible diagnosis of somatic symptom disorder, prompting referral to the psychiatry department. Subsequent standardized psychological assessments confirmed the diagnosis, and targeted psychopharmacological and behavioral interventions led to marked symptom relief after 6 weeks. DeepSeek effectively compensated for the limitations of traditional episodic consultations by maintaining ongoing engagement.

Yuquan District Xicaiyuan Subdistrict Community Health Service Center implemented an AI-powered clinical system based on DeepSeek (26). The system integrated an intelligent knowledge graph covering 7,000 diseases and generated real-time diagnostic recommendations. It accurately identified 39 categories of critical and emergent conditions with an accuracy of 98.6%. Its drug database included over 1,400 medications, and during the trial phase, 136 inappropriate prescriptions were intercepted. AI automatically generated chronic disease follow-up plans, increasing response efficiency for abnormal indicators by 50% and improving public health data aggregation efficiency by 60%. Within 3 months of deployment, the system enhanced performance: outpatient diagnosis compliance reached 94.8%, medical record review time decreased by 70%, and patient satisfaction achieved 98.5% (26).

#### 3.4.2 DeepSeek in health management

DeepSeek leveraged artificial intelligence and big data analytics to identify individuals at high risk for chronic diseases in primary healthcare (21). By integrating data from multiple sources—including wearable devices, telemedicine platforms, and electronic health records—DeepSeek facilitated early screening, risk stratification, and personalized health interventions (21). It constructed dynamic health profiles, monitored patient metrics in real time (e.g., blood glucose and blood pressure), and delivered individualized health guidance through smartphones or wearable devices. Meinian Onehealth used a DeepSeek-powered chatbot to provide diabetes education (27). Longgang District Maternity and Child Healthcare Hospital imported a 2.05-million-word prenatal diagnostic knowledge base into DeepSeek, enabling citizens to access evidence-based prenatal information interactively (28).

DS-Xiaobu Doctor 2.0, an AI-assisted system based on DeepSeek, was evaluated in pediatric outpatient care (29). The system reduced average consultation and diagnostic times to 2.16 and 5.86 min and achieved 92.4% overall diagnostic accuracy (29). Caregiver satisfaction was high, with a Net Promoter Score of +78. In the pediatric pneumonia scenario, DS-Xiaobu Doctor 2.0 (92.4%) outperformed GPT-4Med (89.1%) and Bio MedLM (88.6%) in diagnostic accuracy.

#### 3.4.3 Bridging the urban–rural healthcare gap

DeepSeek enabled rapid deployment of hospital information systems (HIS) within a short period, supporting digital transformation in resource-limited primary care institutions (30). Its AI-assisted diagnostic system enhanced the specialty-level competencies of GPs, addressing challenges of workforce shortages and outdated clinical knowledge (31). In underserved regions, DeepSeek provided essential medical support through remote consultations and intelligent triage. The localized deployment model also improved data security and privacy protection. At the Fifth Affiliated Hospital of Southern Medical University, DeepSeek was used to optimize drug inventory and distribution systems for remote and low-income areas (32). In Yilong County, Nanchong, the system integrated electronic medical records and public health archives, automatically generating individualized “one person, one record” health profiles through dynamic data aggregation and analysis (33). At Youxian People’s Hospital in Mianyang, DeepSeek automatically generated personalized follow-up plans and delivered health reminders to patients (34). On average, each patient encounter saved 15–20 min, resulting in an approximate 30% increase in daily patient throughput for GPs (34).

DeepSeek provided real-time responses to health inquiries, offered guidance on personalized health planning, and delivered preventive recommendations (35). The system supported individuals with varying levels of health literacy through empathetic interactions and extended services to underserved populations. DeepSeek was free and available in multiple formats, including a web version, mobile applications, and a desktop client (11). By enabling scalable access to both health information and emotional support, DeepSeek complemented traditional healthcare delivery and enhanced patient engagement across diverse communities.

#### 3.4.4 DeepSeek in medical education

Generative AI based on DeepSeek reshaped medical education. It enabled the creation of high-fidelity virtual patient cases that simulated the full clinical workflow, including history-taking, physical examination, and diagnosis. Inspur Group Co., Ltd. integrated DeepSeek technology to build diverse simulation scenarios, such as internal medicine emergencies and complex pediatric disorders (36). Through voice interaction and dynamic disease progression, the system trained medical students in diagnostic reasoning and emergency response. It also imitated patients’ emotional expressions and communication needs, helping trainees improve empathy and communication skills (37). At Shandong University QILU Hospital, the AI digital patient system improved the efficiency of standardized resident assessments (38). Using the MoE architecture and DeepSeek’s fine-tuning framework with multimodal reasoning, the time needed to build case-based knowledge databases was reduced by about 30% (38). The platform dynamically generated disease progression pathways, simulated complex clinical decisions, and supported AI-based performance evaluations.

#### 3.4.5 Enhancing general practice research with DeepSeek

DeepSeek’s integrated knowledge base incorporated cutting-edge academic papers, patent databases, and global innovation case libraries, alongside the latest clinical guidelines and typical case examples, addressing the long-standing issue of outdated teaching resources (12, 18). DeepSeek enabled real-time retrieval of academic information, allowing researchers to rapidly access the latest scientific advances. Its data reasoning capabilities assisted general practitioners in various stages of research, including topic selection, study design, data processing, and analysis. DeepSeek efficiently mined large volumes of literature to identify research trends in general practice. It also provided automated literature review generation, experimental design optimization, and innovation assessment, thereby lowering the entry barrier for clinical research. Open-source nature reduced technological barriers, while lightweight model versions (e.g., 1.5B or 7B parameters) enabled cost-effective deployment in primary care institutions (11). When combined with retrieval-augmented generation (RAG), DeepSeek further enhanced the precision and depth of knowledge retrieval and application (39).

### 3.5 Challenges in the application of DeepSeek in primary care

#### 3.5.1 Ethical risks

DeepSeek was susceptible to intrinsic limitations such as algorithmic bias, hallucination, and output unreliability, which raised multiple ethical concerns (40). DeepSeek-R1’s underlying architecture employed a multi-head latent attention mechanism (MLA) to extend contextual processing but retained limitations in long-range semantic modeling (18). This could lead to misinterpretation of complex medical terminology and semantic relationships. The training corpus was primarily derived from open internet sources, inevitably exposing the model to unverified or intentionally manipulated information, a phenomenon often referred to as “data pollution” (41). DeepSeek-R1 also showed biases in decoding metaphorical expressions and culturally embedded emotional content (41). These issues necessitated rigorous pre-deployment validation, human-in-the-loop oversight, and ongoing performance monitoring in clinical settings.

Such technical limitations can translate into real-world ethical dilemmas, as illustrated by a case in which AI-mediated insights challenged professional expertise. A 41-year-old female with a two-year history of a right lung ground-glass nodule (7 mm) actively engaged DeepSeek to analyze longitudinal CT imaging, receiving an AI assessment suggesting high malignancy risk (25). Despite the absence of clinical symptoms and guideline-based recommendations for conservative follow-up, she requested surgical intervention, diverging from the approach initially recommended by her clinicians (25). Postoperative pathology confirmed minimally invasive adenocarcinoma.

#### 3.5.2 Lack of embedded clinical expertise

DeepSeek’s effectiveness depended heavily on the completeness and accuracy of medical data (42). However, electronic medical record data in China has been reported as suboptimal, with high rates of errors in demographic, surgical, and diagnostic information and pervasive issues in interoperability, unstructured entries, and coding inconsistencies (43–45). Such deficiencies could compromise AI systems like DeepSeek by introducing algorithmic bias, reducing diagnostic accuracy, and limiting interpretability. Incomplete or inconsistent real-world data may increase hallucinations, misclassification of medical terms, and errors in semantic inference, ultimately affecting clinical reliability and ethical use. When managing rare diseases (e.g., incidence <1/100,000) or complex cases, the limited availability of relevant data could prevent DeepSeek from accurately capturing disease characteristics, potentially leading to unreliable diagnoses (42). Furthermore, few randomized controlled trials or rigorous clinical validations had been conducted to confirm its clinical effectiveness.

DeepSeek-R1 improved primary care efficiency through lightweight deployment. AI-generated ‘optimal’ solutions represented statistical rather than holistic clinical optimality (46). Patient-specific factors, including psychological traits and cultural context, remained critical in decision-making (47). Continued dependence on AI recommendations without integrating professional expertise could therefore create ethical risks, including potential misdiagnoses and errors in critical clinical decisions (48).

#### 3.5.3 Risk of eroding humanistic care

The increasing adoption of AI systems such as DeepSeek risked diminishing the role of humanistic care in clinical encounters. It could inadvertently reduce meaningful communication and emotional engagement between clinicians and patients (49). For health care professionals, the risks manifest in 2 distinct ways: some clinicians may develop overreliance on DeepSeek, leading to uncritical adoption of its outputs and subsequent diagnostic errors or treatment biases (30, 50). This concern was particularly pronounced in primary care, where continuity, coordination, personalization, and empathy were core service attributes.

When patients access Al-generated medical information without proper clinical context or guidance, they may use these outputs to evaluate the appropriateness of treatments recommended by their physicians. This can generate unwarranted skepticism and new sources of patient–physician conflict. A 54-year-old man had been admitted after routine imaging identified multiple gallstones with radiologic signs of cholecystitis; clinical examination and laboratory tests were unremarkable (25). He underwent an uneventful laparoscopic cholecystectomy and recovered without complication. Following discharge, however, the patient had repeatedly consulted DeepSeek and received conservative-management suggestions (watchful waiting and gallstone-preserving procedures). Citing these AI-sourced opinions, the patient subsequently challenged the surgical indication and filed a formal complaint against the operating team.

This episode illustrated several interrelated concerns. First, LLM outputs could amplify existing guideline ambiguity or present fragmentary evidence that patients may perceive as authoritative (51). Second, when patients acted on AI advice that conflicted with clinician judgment, it often resulted in erosion of clinical authority, breakdowns in informed consent, and new medico-legal disputes (52).

#### 3.5.4 Data privacy and security concerns

DeepSeek’s effectiveness relied on large-scale data collection, raising concerns about privacy and misuse (53). Although regulatory frameworks such as the EU Artificial Intelligence Act and relevant Chinese regulations (15, 16) mandate transparency for high-risk AI systems, including traceable decision pathways, verifiable training data sources, and defined application boundaries (54). DeepSeek-R1 remained vulnerable to privacy breaches, particularly from data theft. Data minimization could partially reduce risks in centralized storage, yet distributed computing nodes remained susceptible to attacks, and data synchronization could be tampered with (41). Knowledge distillation from large to smaller models required feature extraction and transformation, increasing the risk of re-identifying de-identified data. Rapid deployment intensified these challenges, especially in resource-limited healthcare facilities lacking robust cybersecurity (41). Local deployment reduced cloud-related risks but shifted security responsibility to individual institutions, and integration with existing hospital information systems without adequate safeguards could introduce new vulnerabilities, given the model’s extensive parameter requirements and hardware demands (55).

#### 3.5.5 Accessibility and usability across diverse populations

User adaptability varied across demographic groups. Older adults individuals, people with disabilities, and populations with lower educational attainment often faced greater challenges in interacting with AI-driven systems (56). Populations with lower educational attainment or limited health literacy were more likely to misinterpret AI outputs, overlook important recommendations, or struggle to integrate guidance into daily health management (57). These disparities created uneven adoption and utilization of AI systems, potentially exacerbating existing health inequities and limiting the overall effectiveness of digital health interventions in primary care settings (58).

#### 3.5.6 Implementation costs

Deploying AI systems such as DeepSeek in resource-limited primary care settings involves not only direct costs but also substantial indirect expenditures, including hardware requirements (high-performance servers, secure data storage, reliable internet), staff training, and technical support (50).

### 3.6 Practical recommendations for implementing DeepSeek in primary care

#### 3.6.1 Data mining and knowledge graph construction

To enhance DeepSeek’s interpretability and clinical relevance, primary care institutions should integrate multi-source healthcare data—including electronic health records, laboratory results, imaging studies, and wearable device data—into standardized, structured databases. Clinical knowledge graphs linking diseases, symptoms, treatments, and patient characteristics can then be constructed to provide semantic context. These knowledge graphs should be continuously updated with newly published guidelines and local clinical protocols. Fine-tuning DeepSeek on these graphs allows the model to generate evidence-based, transparent recommendations tailored to individual patients, enabling reproducible and clinically meaningful decision support.

Effective deployment of DeepSeek required deep integration of domain-specific medical knowledge (30). To ensure that model outputs aligned with real-world clinical practice, the development and interpretation of LLMs needed involvement from healthcare professionals with general practice expertise (40). Embedding clinical reasoning into model design was essential to improve interpretability and clinical relevance, particularly in primary care, where decision-making was multifactorial and highly individualized.

#### 3.6.2 Clinical validation

Robust evaluation of DeepSeek’s clinical effectiveness requires well-designed multicentre studies. Primary care networks should implement pre–post intervention designs or randomized controlled trials comparing outcomes with and without DeepSeek assistance. Data collection should encompass diagnostic accuracy, treatment adherence, workflow efficiency, and patient-reported outcomes such as satisfaction and engagement (59). The findings can guide iterative model refinement, validate AI-driven recommendations in real-world settings, and provide a scientific basis for scaling deployment across diverse primary care environments.

To enhance inclusiveness, DeepSeek should support multimodal human-computer interaction, including voice commands, touch interfaces, and gesture recognition, alongside assistive features tailored to specific populations. Incorporating multilingual and localized content could mitigate comprehension issues arising from regional dialects or speech impairments, reducing risks of misinterpretation and diagnostic error (60).

#### 3.6.3 Training programs for general practitioners

Successful adoption of DeepSeek depends on comprehensive clinician training. Health systems should establish structured programs that combine hands-on workshops, online modules, and case-based simulations to teach general practitioners how to interpret AI outputs, verify recommendations, and integrate insights into patient management. Training should include strategies for identifying model limitations, mitigating overreliance on AI, and maintaining professional judgment. Periodic assessments and refresher courses can ensure sustained proficiency and safe application of AI-assisted clinical decision-making. Developers were encouraged to deliberately integrate humanistic elements into AI systems to address patients’ emotional, psychological, and social needs, thereby preserving doctor–patient rapport (61).

#### 3.6.4 Ethical and technical guidelines

To ensure responsible use, healthcare institutions should establish clear ethical and technical guidelines for DeepSeek deployment. These should define the boundaries for recommendation adoption, affirming that final clinical decisions rest with the physician. Policies must address data privacy, security, and governance, including protocols for handling sensitive patient information. Additionally, mechanisms for monitoring model performance, detecting biases, and ensuring fairness and transparency should be implemented (62). Clear documentation of model updates and decision logic will further support accountability and maintain trust in AI-assisted primary care.

#### 3.6.5 Implementing a closed-loop primary care model with DeepSeek

**Figure 3:**
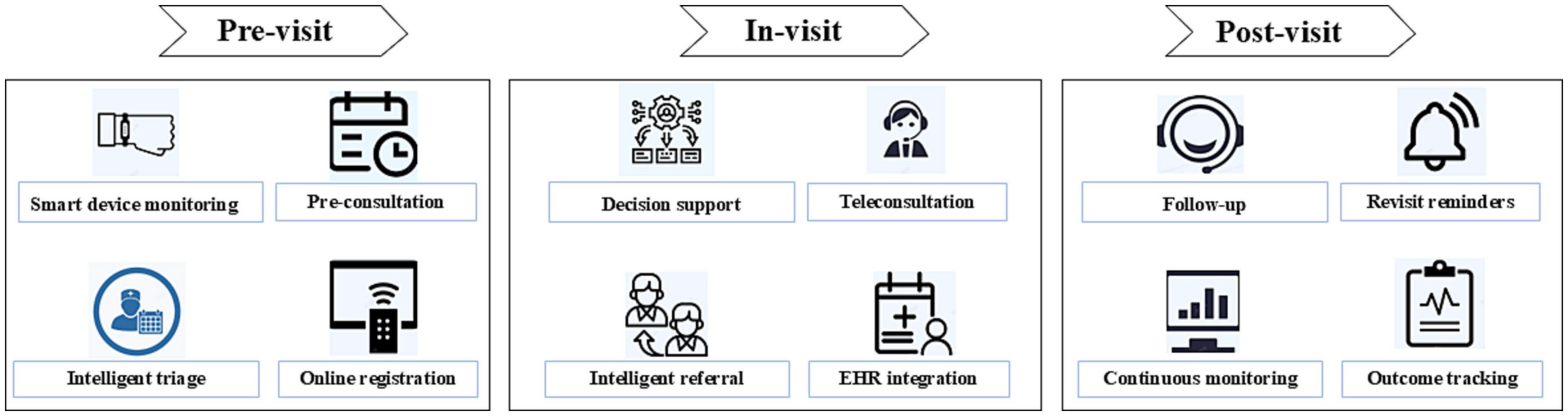
Digital health–enabled primary care workflow across pre-visit, in-visit, and post-visit stages.

DeepSeek supported a closed-loop primary care model, comprising pre-visit AI-assisted triage and data collection, in-visit clinical decision support and teleconsultation, and post-visit follow-up with automated reminders and continuous monitoring (23). Its intelligent health management system incorporated the latest clinical evidence and guidelines, enabling adaptive and individualized care, particularly for chronic disease management in community populations (23). The platform delivered targeted health education and real-time care recommendations, which promoted healthier lifestyles and facilitated timely clinical interventions (Figure 3) (23).

## 4 Strengths and limitations

This review summarized the application of DeepSeek in Chinese primary care, covering clinical practice, research, education, and health management. It provided actionable recommendations for implementation. However, few original studies or multicenter trials directly evaluated its clinical effectiveness, particularly in real-world primary care settings. Most data came from pilot projects or institutional reports, limiting generalizability and the assessment of long-term impacts on patient outcomes and workflow efficiency.

## 5 Conclusion

DeepSeek demonstrated considerable potential in supporting primary care in China by enhancing clinical decision-making, chronic disease management, medical education, and research productivity. It was currently applied in primary care settings, where it assisted clinicians, supported health management, and facilitated training and research. However, challenges remained, including limited interpretability, potential reduction in humanistic care, accessibility issues, technical limitations, and data privacy concerns. To maximize the benefits of DeepSeek, future efforts should focus on rigorous clinical validation, structured training for general practitioners, robust ethical and technical guidelines, and integration with local healthcare data and knowledge systems. By addressing these challenges, DeepSeek and similar AI tools can empower primary care providers, promote equitable healthcare delivery, and contribute to a more intelligent and patient-centered healthcare system.
